# Augmented anticancer effect and antibacterial activity of silver nanoparticles synthesized by using *Taxus wallichiana* leaf extract

**DOI:** 10.7717/peerj.14391

**Published:** 2022-11-23

**Authors:** Aliya Yousaf, Muhammad Waseem, Aneela Javed, Sofia Baig, Bushra Ismail, Ayesha Baig, Irum Shahzadi, Shamyla Nawazish, Iftikhar Zaman

**Affiliations:** 1 Department of Biotechnology, COMSATS University Islamabad, Abbottabad Campus, Abbottabad, Pakistan; 2 Department of Chemistry, COMSATS University Islamabad, Islamabad Campus, Islamabad, Pakistan; 3 Department of Healthcare Biotechnology, Atta ur Rahman School of Applied Biosciences (ASAB), National University of Sciences and Technology (NUST), Islamabad, Pakistan; 4 Department of Environmental Sciences, Institute of Environmental Sciences and Engineering (IESE), National University of Sciences and Technology (NUST), Islamabad, Pakistan; 5 Department of Chemistry, COMSATS University Islamabad, Abbottabad Campus, Abbottabad, Pakistan; 6 Department of Environmental Sciences, COMSATS University Islamabad, Abbottabad Campus, Abbottabad, Pakistan; 7 Zoo/Wildlife Conservation, Peshawar, Khyber Pakhtunkhawa, Pakistan

**Keywords:** *Taxus wallichiana*, Nanoparticles, Anticancer, Antibacterial acticity

## Abstract

**Background:**

*Taxus wallichiana* is an evergreen tree species found in the Himalayan region of Pakistan. The tree possesses important secondary metabolites such as Taxol that has been implicated in treating breast, ovarian and colon cancer. Therefore keeping in view the importance of this plant species, silver nanoparticles were synthesized using *Taxus wallichiana* aqueous leaf extract and evaluated for their anti-bacterial and anti-cancer properties.

**Methods:**

Silver (Ag) nanoparticles (NPs) were characterized for their optical, morphological and structural features using techniques such as UV-visible spectroscopy, X-ray diffraction (XRD), Fourier transform infrared (FTIR) spectroscopy, scanning electron microscopy (SEM) and energy dispersive X-ray (EDX) and were evaluated for their antibacterial activity and anti-cancer activity using U251 cell line.

**Results:**

The study showed that the UV-absorbance peak of Ag_2_O NPs at 450 nm shifted to 410 nm, affirming the formation of leaf extract Ag NPs. Similarly structural studies revealed the crystalline nature of the cubic structure of the Ag crystal with an average crystallite size of 29 nm. FTIR analysis exhibited the existence of different functional elements including O-H and N-H and phenolic groups. Non-spherical glomerular shaped *Taxus wallichiana* Ag NPs were observed from SEM studies and EDX profile showed Ag as the main element along with constituent of biological origin. The synthesized Ag NPs showed significant antibacterial activity against *Salmonella typhi*, and *Staphylococcus aureus*. The cytotoxic activity of Ag NPs on U251 brain cancer cells showed a synergistic effect with 10 ug/mL concentration after 48 and 72 h incubation based on cell viability assay indicating promising glioblastoma drug potential.

## Introduction

Nanotechnology involves using synthesized nanoparticles ranging from 1–100 nm in size. As the ratio of surface area to volume increases, the particle size decreases and as a result, physical, chemical and biological properties of these particles alter significantly compared to the wholesome particles (Dos Santos et al., 2014; Narayanan & Sakthivel, 2013) Nanoparticles are extensively used for medical purpose, disease management and environment conservation (Ebrahimzadeh et al., 2020). Metal nanoparticles such as Au, Cu, Zn, Fe and Ag have received wide range of applications due to their physical, catalytic and optical properties and have been utilized in diverse fields such as therapeutics and electronics (Lee & Jun, 2019). The synthesis of silver nanoparticles (Ag NPs) by using easy, sustainable, cost effective and ecofriendly approaches have received greater attention in the recent years (Keat et al., 2015). Ag NPs have particularly shown promising results for anticancer, anti-inflammatory and wound healing properties. These particles have also been utilized for their antimicrobial characteristics (Singh et al., 2016). Numerous methods including electro-irradiation, laser mediated, and photochemical treatment are being used for Ag NPs synthesis, but they are mostly expensive and cause toxicity in the environment (Li & Zhang, 2010). In contrast to this, biological based methods using aqueous plant or microbial extracts are preferred (Rafique et al., 2017). Biological process for the synthesis of Ag NPs by using plant extract is useful due to its less toxicity, compatibility and longer synthesis time with narrow particle size distribution (Hema et al., 2016). Large numbers of secondary metabolites are present in plant extracts that act as reducing agents and play an important role in capping and stabilizing of Ag NPs (Shah et al., 2019). *Peaonia emodi*, *Althaea rosea*, *Swertia chirata*, *Bergenia stracheyi* and *Solanum xanthocarpum* are some important medicinal plants with secondary metabolites having pharmaceutical importance (Shah et al., 2019; Korbekandi et al., 2016; Kumar & Sinha, 2017; Rashid et al., 2019; Amin et al., 2012). Ag NPs were synthesized from different parts of these plant species such as root, leaf, flower and fruit having immense pharmaceutical significance (Korbekandi et al., 2016; Kumar & Sinha, 2017; Rashid et al., 2019; Amin et al., 2012).

*Taxus wallichiana* is an important tree species that is present in the northern areas of Pakistan including Nathia Gali, Thandianai and Gilgit Baltistan. *Taxus wallichiana* is traditionally used for the treatment of asthma, bronchitis, cough, diarrhea, inflammation and headache (Sinha, 2020). Furthermore, its leaf extract possess antioxidant, antimicrobial, anti-inflammatory, anti-pyretic and analgesic properties (Nisar et al., 2008). These properties are mainly due to the presence of secondary metabolites such as diterpetens, flavonoids, steroids and lignins. *Taxus wallichiana* is extremely important tree species because of its anti-cancerous property attributed to alkaloid Taxol or Paclitaxel (Adhikari et al., 2022). Additionally, many phytochemicals like toxoids, flavonoids, lignans, sugars and steroids derivatives have been found in several species of *Taxus* including *Taxus wallichiana*. It was observed that plant based silver nanoparticles show very potent anticancer activity. This is due to metabolites adsorbed on silver nanoparticles. The synergistic effect of silver ions and plant based metabolites could be the reason for higher anticancer activity. Biocompatibility and toxicity of plant-based Ag NPs is the emerging new era in therapeutic diagnostics. Plant mediated silver nanoparticles exhibited anti-cancer activity against various cancer cell lines such as breast cancer MCF-7, MDA-MB-231 and brain cancer U251 cells (Zhao et al., 2020; Wypij et al., 2021; Oves et al., 2022).

Similarly pathogenic bacteria are becoming increasingly resistant to drugs available in the market by ever evolving strategies based on microbe associated molecular patterns (MAMPs), effector triggered immunity and transcriptional reprogramming. Treatment of various pathogenic diseases though medicinal plants is as old as human civilization. Thus, important metabolites associated with plant extract makes plant-based Ag NPs an effective strategy against pathogenic bacteria which are becoming resistance to very high level of antibiotics. Ag NPs can easily reach cells and interact with cell constituents and disrupt cell signaling pathways based on oxidative enzymes manipulation, DNA damage or reactive oxygen species (ROS) production. Thus altogether, plant-based Ag NPs have the potential to become new antibiotics against bacterial pathogen and can provide major breakthrough in cancer therapeutics.

Based on this, the current study is aimed at the synthesis and characterization of *Taxus wallichiana* Ag NPs which were also tested for their anti-microbial and anticancer properties. Ag NPs synthesis was confirmed using XRD, SEM, FTIR and EDX. To the best of our knowledge, this is the first report of *Taxus wallichiana* aqueous leaf-based Ag NPs being used for anticancer activity against U251 human malignant glioblastoma multiforme cell line and evaluated against gram-positive and gram-negative pathogenic bacteria.

## Materials and Methods

Silver oxide, ammonium hydroxide and sodium borohydride were acquired from Sigma Aldrich (Germany). *Taxus wallichiana* leaves used in this experiment were collected from the northern areas of Pakistan, which include Nathia Gali and Donga Gali.

### Preparation of leaf extract

Fresh leaves of *Taxus wallichiana* were collected, and washed with distilled water, dried in shade and grinded to fine powder. To prepare leaf extract of *Taxus wallichiana*, 20 g of leaf powder was taken and added into 200 mL of deionized distilled water. By using magnetic stirrer, the mixture was boiled at 60 °C for 30 min. The extract was then filtered for nanoparticles synthesis (Ahmed et al., 2016).

### Synthesis of silver nanoparticles

**Figure 1:**
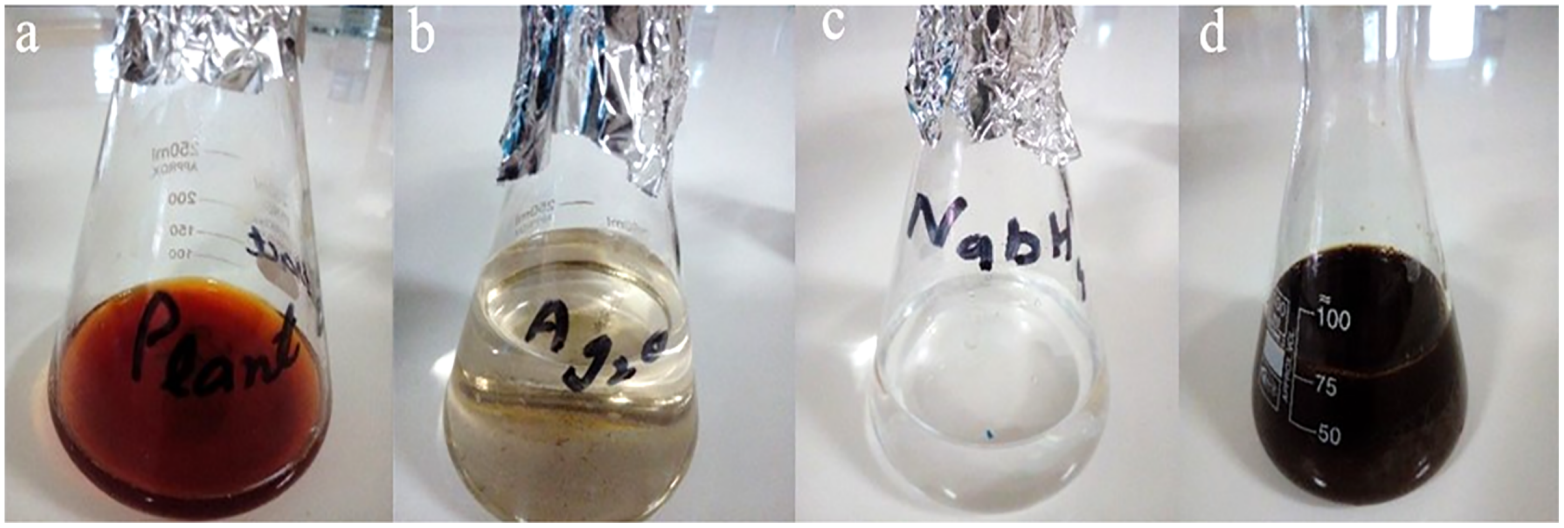
Synthesis of *Taxus wallichiana* Ag NPs. (A) *Taxus wallichiana* leaf extract, (B) silver oxide, (C) sodium borohydride, (D) synthesis mixture of Ag NPs.

Stock solution of 10 mM of silver oxide and 5 mM of sodium borohydride were initially prepared. For making nanoparticles, 25 mL of plant extract was taken in the flask to which 25 mL of silver oxide along with sodium borohydride (25 mL) was added. The change in color was observed (Fig. 1), after which the flask was placed on hot plate with continuous stirring for 30 min with the addition of ammonium hydroxide. After 30 min flask was placed at room temperature for 24 h (Rashid et al., 2019). To separate Ag NPs from liquid solution, centrifugation was done at 12,000 rpm for 10 min at 25 °C. The stable Ag NPs were washed three times with the deionized distilled water to enhance Ag NPs refinement. Ag NPs were then dried and stored carefully in a bottle for future analysis (Shah et al., 2019; Shehzad et al., 2018).

### Characterization

#### UV-spectroscopy

Bio-reduction of Ag NPs was observed by ultraviolet–visible (UV–Vis) spectroscopy (UV-1602, BMS). UV spectrum with plant extract Ag NPs in 10 ml of solution was monitored. The UV–Vis absorption spectra for Ag NPs was observed in a range of 300–800 nm with UV-1602 spectrophotometer (Bhat et al., 2021).

#### X-ray diffraction (XRD)

XRD facilitates the recognition of crystalline structure of Ag NPs. XRD was performed with Cu radiation at an angular range of 2θ = 10°–80°. The air dried and centrifuged Ag NPs were placed on glass slide and studied using XRD (BRUKER D8 X-ray diffractometer). The average crystallite sizes of samples were calculated using Scherrer’s formula:

$\rm{D}={\rm K} {\lambda} /\beta1/2\,cos\theta$

D represents size of the particle, K is constant, λ is wavelength, β is full width at half maxima and θ is Bragg’s angle. The acquired diffraction patterns were plotted by using Origin software to obtain the structural information about the samples (Shehzad et al., 2018, Rashid et al., 2019).

#### Scanning electron microscopy (SEM) and energy dispersive X-ray (EDX) spectroscopy

Analysis of the shape and size of synthesized Ag NPs was done by using SEM. The micrographs were obtained by coating the powder with gold and analyzed by using Philips XL30 model electron microscope at a voltage 20 kV under vacuum pressure (5–10 torr). EDX, a technique that defines the elemental composition of the sample was used to confirm the presence of silver as well as elemental composition of other elements in synthesized *Taxus wallichiana* Ag NPs (Shahzadi et al., 2022).

#### Fourier transform infrared spectroscopy (FTIR)

The most important functional groups which are involved in the synthesis of silver nanoparticles were identified by means of FTIR spectroscopic technique. The FTIR spectra were recorded in the wave number range between 4,000 and 400 cm^−1^ (Bhat et al., 2021; Reda et al., 2019).

### Anti-bacterial activity

To assess the antibacterial activity of synthesized Ag NPs, aqueous extract of *Taxus wallichiana* and Ag_2_O NPs were used against *Escherichia coli* (ATCC 10536), *Pseudomonas aeruginosa* (ATCC 9027), *Salmonella typhi* (ATCC 6539) and *Staphylococcus aureus* (KX262679) bacterial strains (Rashid et al., 2019). To evaluate the antibacterial activity of the plant extract, Ag_2_O and *T. wallichiana* synthesized Ag NPs, Agar well diffusion method was used. For bacterial growth, nutrient broth medium (Oxoid, Hampshire, England) was prepared and autoclaved at 121 °C for 15 min. For the preparation of inoculum, 24-h bacteria culture was grown in nutrient broth and 0.5 OD was maintained using spectrophotometer of 600 nm wavelength (BMS UV-1602) (Rashid et al., 2019; Shah et al., 2019).

Nutrient agar (Oxiod, Hampshire, England), plating medium was prepared. Nutrient agar was autoclaved at 121 °C for 15 min and poured in petri plates (Shahzadi et al., 2022). The selected bacterial cultures were inoculated individually on petri plates. For the preparation of well, a 6-mm sterile metallic borer was used. Three different concentrations of Ag NPs were used including 30 (μg/well), 60 (μg/well) and 90 (μg/well) with Ag_2_O NPs concentaron of 30 ug/well. Ampicillin 30 (ug/well; Sigma Aldrich, St. Louis, MO, USA) was used as a positive control and water was used as a negative control. The petri plates were kept in incubator for 24 h at 37 °C. The diameter of the clear zones was measured around each well as a zone of inhibition (mm). All tests were performed in triplicate (Shahzadi et al., 2022).

### Cell viability assay

#### Cell culture

U251 cells, derived from a human malignant glioblastoma multiforme (GBM) were incubated with MTT (3-[4,5- dimethylthiazol-2-yl]-2,5-diphenyltetrazolium bromide) (Sigma Aldrich, St. Louis, MO, USA) and 1% penicillin/streptomycin in Dulbecco modified eagle media (DMEM) containing extra glucose and 10% Fetal Bovine Serum (FBS; Thermo Fischer Scientific, Waltham, MA, USA). Cells were maintained and upon reaching 90 percent confluence, trypsinised and sub cultured until needed for MTT assay (Arshad et al., 2022).

#### *In* vitro cytotoxicity assay (MTT assay)

Exponentially growing U251 brain cancer cells were counted and 10,000 cells per well were plated, in triplicate, in 96-well plates (Nunc, Roskilde, Denmark). The cells volume was kept at 100 μL per well. Stock solutions of *Taxus wallichiana* leaf extract, Ag NPs and Ag_2_O NPs were prepared in sterile distilled water and diluted to different test concentrations (1.25, 2.5, 5, 10, and 20 μg/mL) with cell supplemented media of ~200 μL/well as final volume. Each concentration was tested in triplicate on U251 cells. Control wells blank media (without cells) and solvent control (without drug). MTT solution of 15 μL was added to each well and incubated for 3 h at 37 °C. MTT solution was initially prepared by dissolving 5 mg/mL in 1 mL of Phosphate buffered saline (PBS; Thermo Fisher Scientific, Waltham, MA, USA). Intracellular purple formazan crystals became visible under microscope after incubation of U251 cells with MTT solution. The solution was removed from each well after the formation of formazan crystals. 150 uL of dimethyl sulfoxide (DMSO; Sigma Aldrich, St. Louis, MO, USA) was added in each well as a solubilizing agent (Arshad et al., 2022). Finally, the absorbance of the cells was measured by using spectrophotometer at 550 nm. *Taxus wallichiana* leaf extract, Ag NPs and Ag_2_O NPs were tested separately for percent cytotoxicity after 48 and 72 at different concentrations. The influence of the formulations on the cell viability was determined by formula:

% Viability = (A570 of the treated cells − A570 of the control cells – A570 of the blank cells) × 100.

Cells pictures in all wells were taken at different concentrations and time points using 10 × Nikon TS 100 Microscope before the addition of MTT reagent (Naveed et al., 2022).

### Statistical analyses

The data was analyzed by R software (R Core Team, 2022) for antimicrobial and anticancer activity. Triplicate measurements were averaged with standard error (±SE). One-way and Two-way analysis of variance (ANOVA) was performed at *p* < 0.05 followed by least significant difference (LSD) test for antimicrobial and anticancer activity respectively.

## Results and discussion

The main reason of our study was to synthesize Ag NPs based on *Taxus wallichiana* leaf extract and use them against bacterial pathogen and in cancer therapeutics. Silver nanoparticles were synthesized by heating and stirring mixture of aqueous silver oxide solution and *Taxus wallichiana* leaf extract constantly. After a while, yellow color reaction mixture gradually changed into a dark-brown suspension. This showed that silver ions in the reaction have been transformed into silver (Shah et al., 2019). Thus, synthesis of Ag NPs was done successfully through less harmful procedure as the process did not require much refinement as compared to other methods (Niraimathi et al., 2013). Synthesis of silver nanoparticles by using the plant extract is not completely understandable but still in the reduction process a combination secondary compounds are expected to be the primary role in their synthesis (Priya, Vijayakumar & Janani, 2020). According to numerous studies proteins and enzymes are also involved e in the synthesis of silver nanoparticles (Sasidharan et al., 2020).

### UV-visible spectroscopy

**Figure 2:**
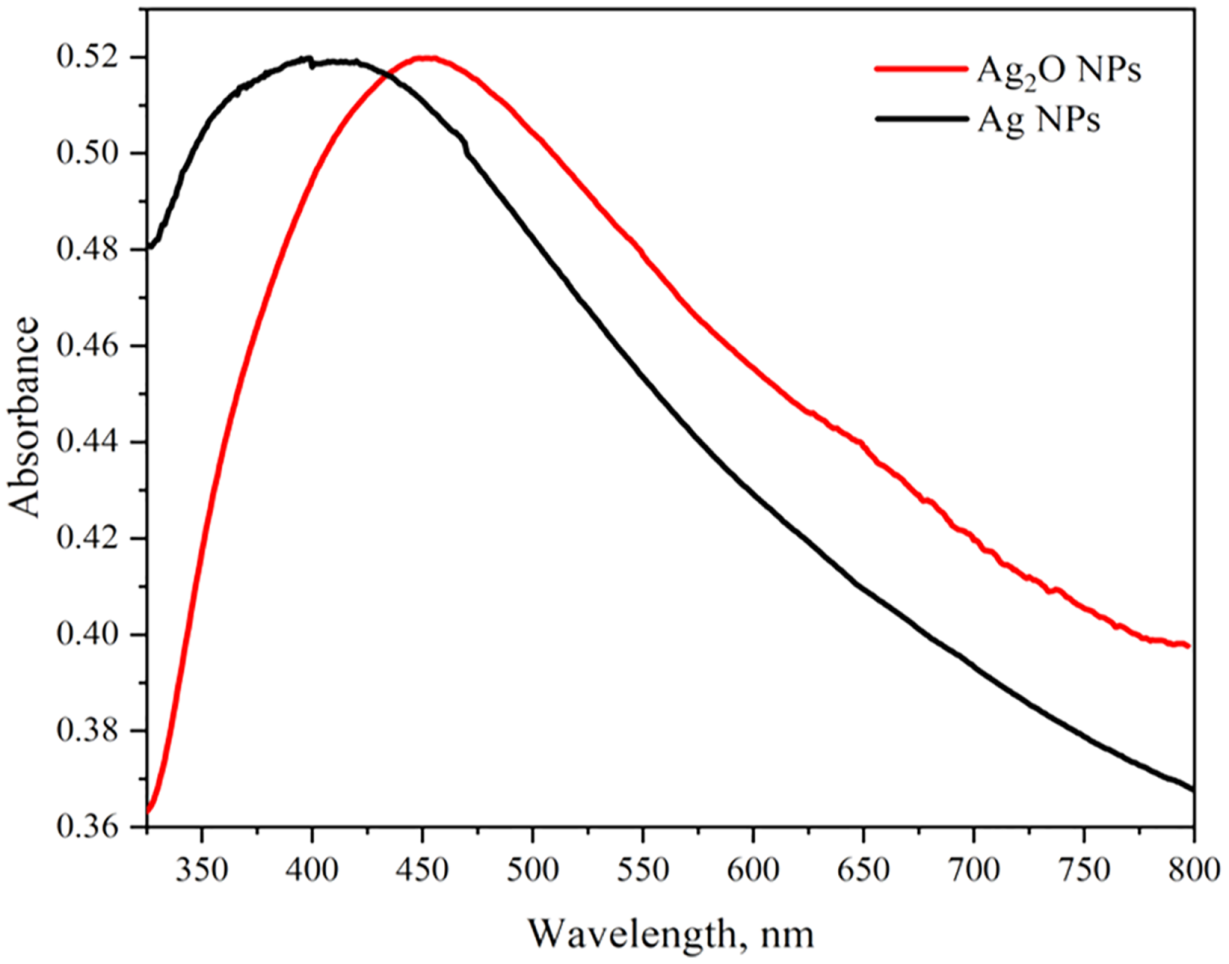
UV-Vis spectra of Ag_2_O NPs and Ag NPs.

**Figure 3:**
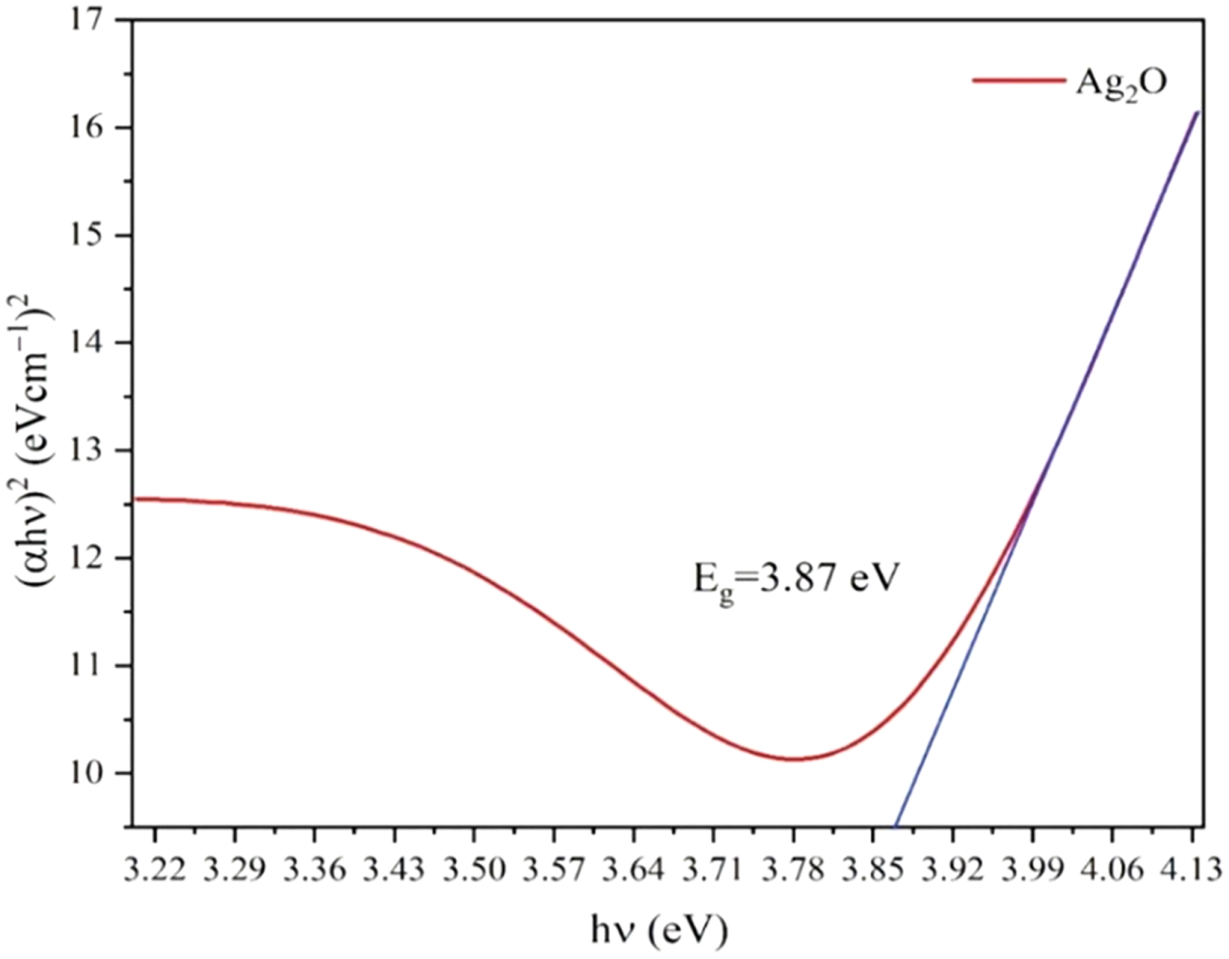
Tauc’s plots for the band gap energy of Ag_2_O NPs.

The optical properties of Ag_2_O NPs and Ag NPs were determined at room temperature by UV-Visible Spectrophotometer Lambda 25 model (Perkin Elmer, Waltham, MA, USA) between 300 to 800 nm. The UV-vis spectra of Ag NPs and Ag_2_O NPs are represented in Fig. 2. The UV absorption peak for Ag_2_ONPs appeared at 450 nm which shifted to the lower wavelength after change in a color from yellow to dark brown that confirmed the reduction of Ag_2_O NPs to Ag NPs. The characteristic surface plasmon resonance (SPR) peak for Ag NPs appeared at 410 nm. This result is in agreement with the findings reported by Okafor et al. (2013) while studying the plant mediated synthesis of silver nanoparticles and the blue shift further confirmed the successful conversion of Ag_2_O to Ag nanoparticles. The presence of bioactive compounds in *Taxus wallichiana* plant extract were found effective to reduce the silver to it to nano-size. The optical band gap of Ag_2_O NPs was calculated by using Tauc’s relation (Sirohi & Sharma, 1999). The band gap of Ag_2_O NPs was found 3.87 eV. The band gap energy value of Ag NPs was found more than previously value reported in literature which may be due to quantum confinement (Fig. 3; Das et al., 2016).

### XRD

**Figure 4:**
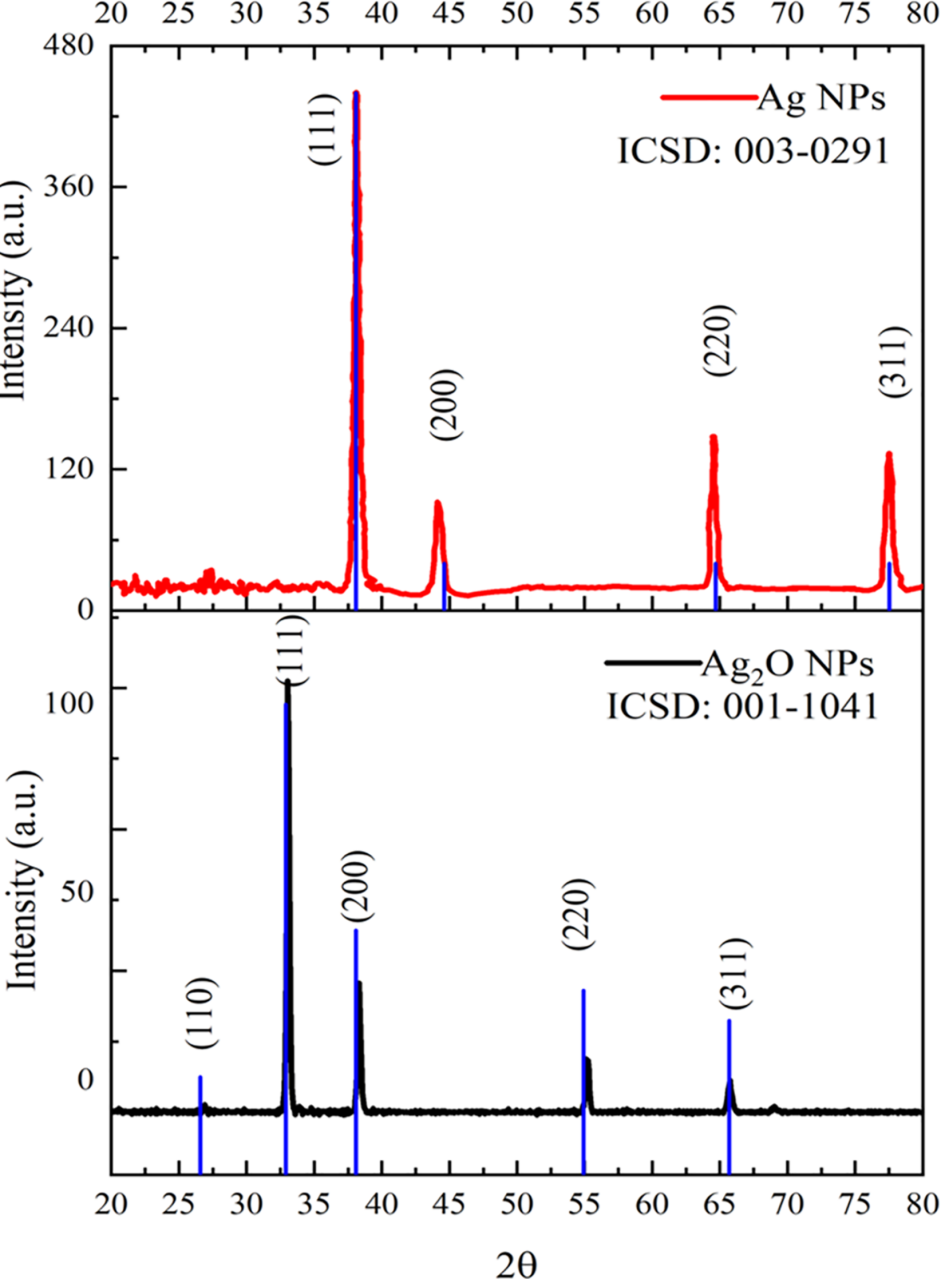
XRD patterns of Ag and Ag_2_O NPs.

The diffractogram of Ag NPs and Ag_2_O NPs is represented in Fig. 4. The XRD pattern of synthesized Ag NPs by the reduction of silver ions with aqueous extracts of *Taxus wallichiana* leaves shows the distinctive peaks in the spectrum with 2θ values ranging from 20°–80°. Indexing of the peaks was carried out by comparing with the standard pattern of cubic Ag (ICSD: 003-0291). The diffraction pattern of *Taxus wallichiana* leaf extract-based Ag NPs displayed four strong peaks at 2θ values 38.06°, 44.25°, 64.51° and 77.44° which can be indexed to (111), (200), (220), and (311) planes (ICSD: 001-1041). This shows that silver is the main component in *Taxus wallichiana* based nanoparticles. The crystal structure of synthesized silver nanoparticles was face centered cubic. The size of the AgNPs was found 29 nm as estimated from full width at half maximum (FWHM) of all the four peaks by using Scherer’s formula. The average crystallite size of Ag_2_O NPs calculated from XRD data was 26 nm.

### SEM/EDX

**Figure 5:**
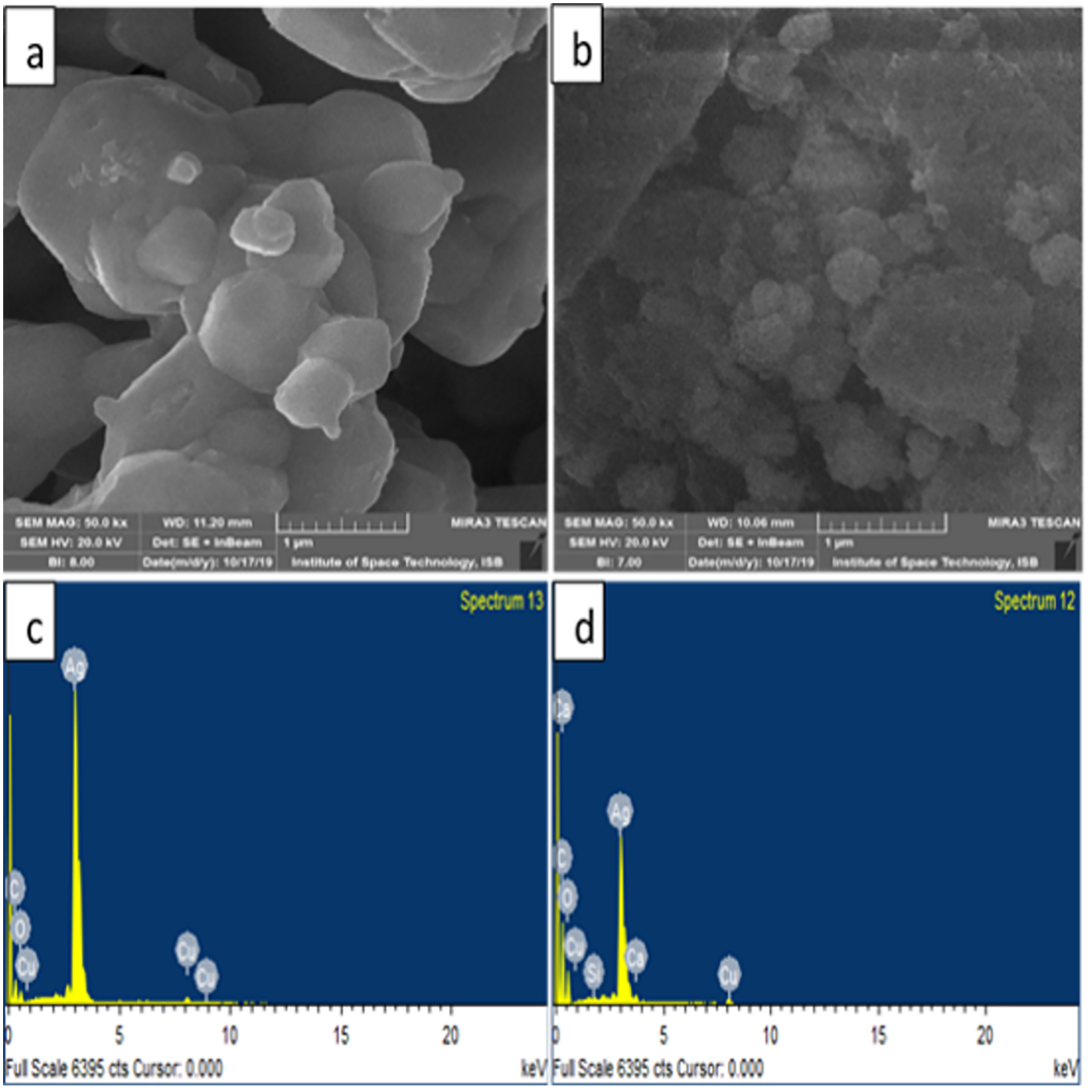
SEM images of (A) Ag_2_O NPs (B) Ag NPs (C) EDX of Ag_2_O NPs (D) EDX of Ag NPs.

Figure 5 represents the microscopic structure of synthesized silver nanoparticles and silver oxide nanoparticles. The micrograph displayed that silver oxide particles are nearly spherical shaped, and their size varied from 200–1,000 nm (Fig. 5A). On the other hand, the size of silver particles was found less than 1,000 nm (Fig. 5B). However, due to aggregation, it is difficult to measure the exact size of particle (Sasidharan et al., 2020). Elements composition present in the sample was shown with the help of EDX analysis. The signals thus obtained confirm C, Cu, O, Ca, and Si presence in silver nanoparticles. EDX analysis of silver oxide nanoparticle indicated the presence of silver with a weight percentage of 83.4% (Fig. 5C) while in case of Ag NPs it was 48.9% by weight (Fig. 5D). This indicates the presence of more silver in Ag_2_O NPs than Ag NPs which is in accordance with their molecular formula. In both samples, EDX showed strong peaks for silver which confirmed their presence as a major constituent of nanoparticles. The presence of other elements was due to macronutrients such as calcium present in the plant extract. The existence of other elements in the smaller quantities was due to their incorporation during the synthesis process.

### FTIR spectroscopy

**Figure 6:**
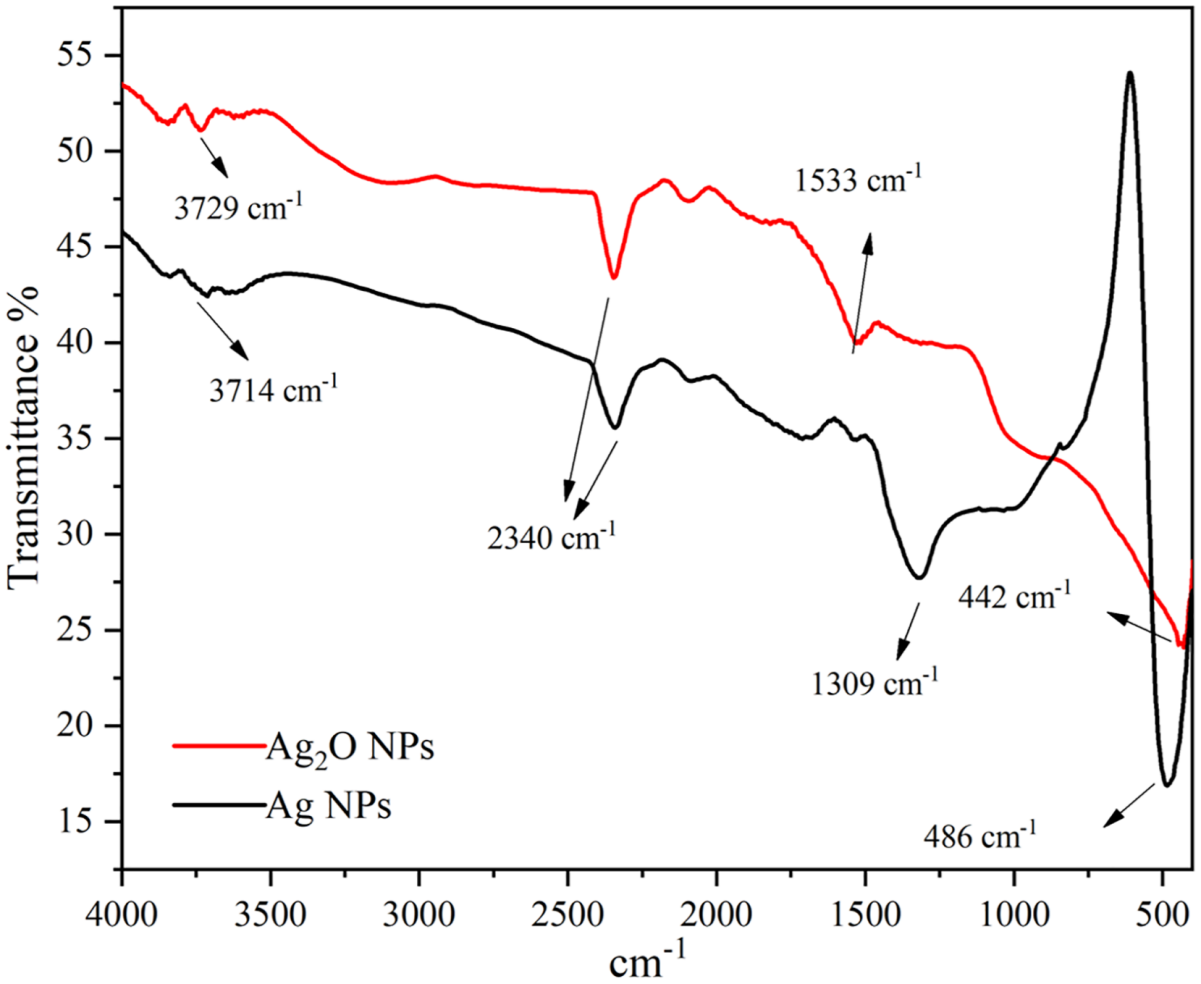
FTIR spectra of Ag and Ag_2_O NPs.

The presence of functional groups in the Ag NPs as extracted from *Taxus wallichiana* and Ag_2_O NPs were identified and shown in Fig. 6. The FTIR of Ag_2_O NPs showed diverse stretching bands that appeared at 442 cm^−1^, 1,533 cm^−1^, 2,340 cm^−1^ and 3,729 cm^−1^. While, Ag NPs showed peaks at 486 cm^−1^, 1,309 cm^−1^, 2,340 cm^−1^ and 3,714 cm^−1^ and matched well with the published literature (Rashmi et al., 2020). Presence of aromatic amino group absorption band at 1,309–1,310 cm^−1^ signifies C-N stretching (Mandal et al., 2021). Whereas a peak at 1,533 cm^−1^ corresponds to C=C stretching of alkene and aromatic ring (Afreen et al., 2020). A peak appeared at 2,340 cm^−1^ is assigned to NH_3_ structure assigned to amino group stretching (Gopinath et al., 2015). The presence of these functional groups played significant role in the capping of Ag NPs. The characteristic bands obtained in the spectrum of Ag_2_O NPs were also found useful in capping and stabilizing the particles (Fig. 6). A broad band in the range 3,200–3,400 cm^−1^ appeared only in the spectrum of Ag_2_O NPs showed that silver in oxide form was more hydrated than bare silver nanoparticles. Peaks in the range of 3,200–3,700 cm^−1^ were assigned as stretching of N-H group and OH stretching in alcohols and phenolic compounds with strong hydrogen bonds (Helmy et al., 2020). Small intense peaks at 3,714 cm^− 1^ and 3,729 cm^− 1^ denote O-H stretching vibrations. This may denote stretching vibrations of alcohols and phenolic groups from diterpines and lignans in Ag NPs (Ajitha et al., 2016). In plant extract these functional groups are present due to amine group, flavonoids and alcohols that are responsible for the reduction of silver in Ag NPs.

### Antibacterial activity of Ag NPs

**Figure 7:**
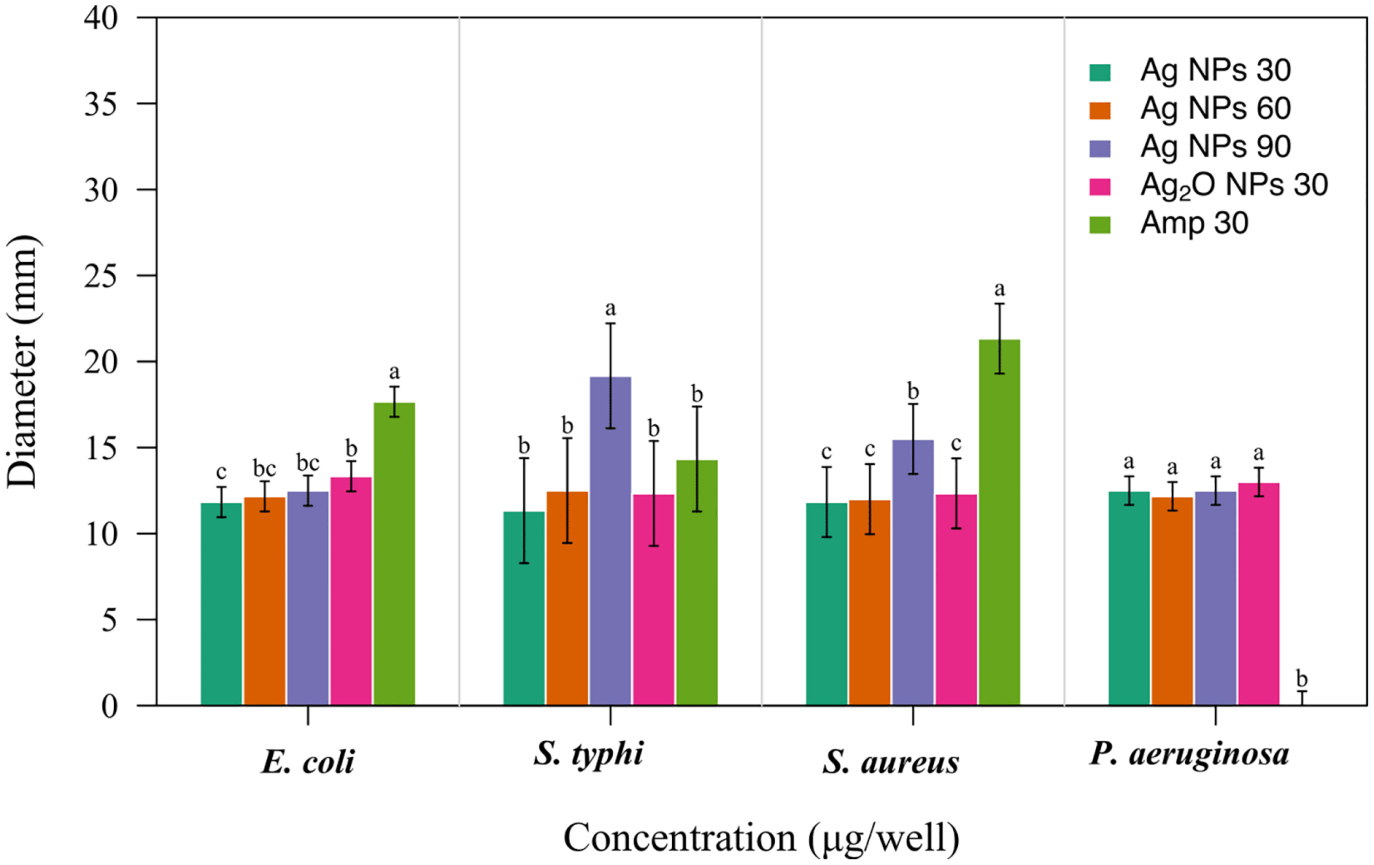
Antibacterial activity of *Taxus wallichiana* Ag NPs. Bacterial zone of inhibition with Ag NPs at 30, 60 and 90 ug/ul and Ag_2_O NPs at 30 ug/ul against (A) *Escherichia coli* (B) *Salmonella typhi* (C) *Staphylococcus aureus* (D) *Pseudomonas aeruginosa*. Ampicillin is used as positive control. Data is represented as means ± SE from three replicates. Different letters represent significant differences with Fisher LSD test at *p* ≤ 0.05.

Pathogenic bacteria are the major cause of infectious diseases that pose a serious threat to human population. Medicinal plants and their various organ parts have been used to counter such pathogenic bacteria from ancient times and in many parts of the world (Sökmen et al., 2017). In this study, *Taxus wallichiana* Ag NPs were used against bacterial pathogens (Fig. 7). This the first study in which Ag NPs synthesized by using the aqueous leaf extract of *Taxus wallichaina* were tested against *Escherichia coli* (*E. coli*), *Pseudomonas aeruginosa* (*P. aeruginosa*) *Salmonella typhi* (*S. typhi*) and *Staphylococcus aureus* (*S. aureus*).

**Figure 8:**
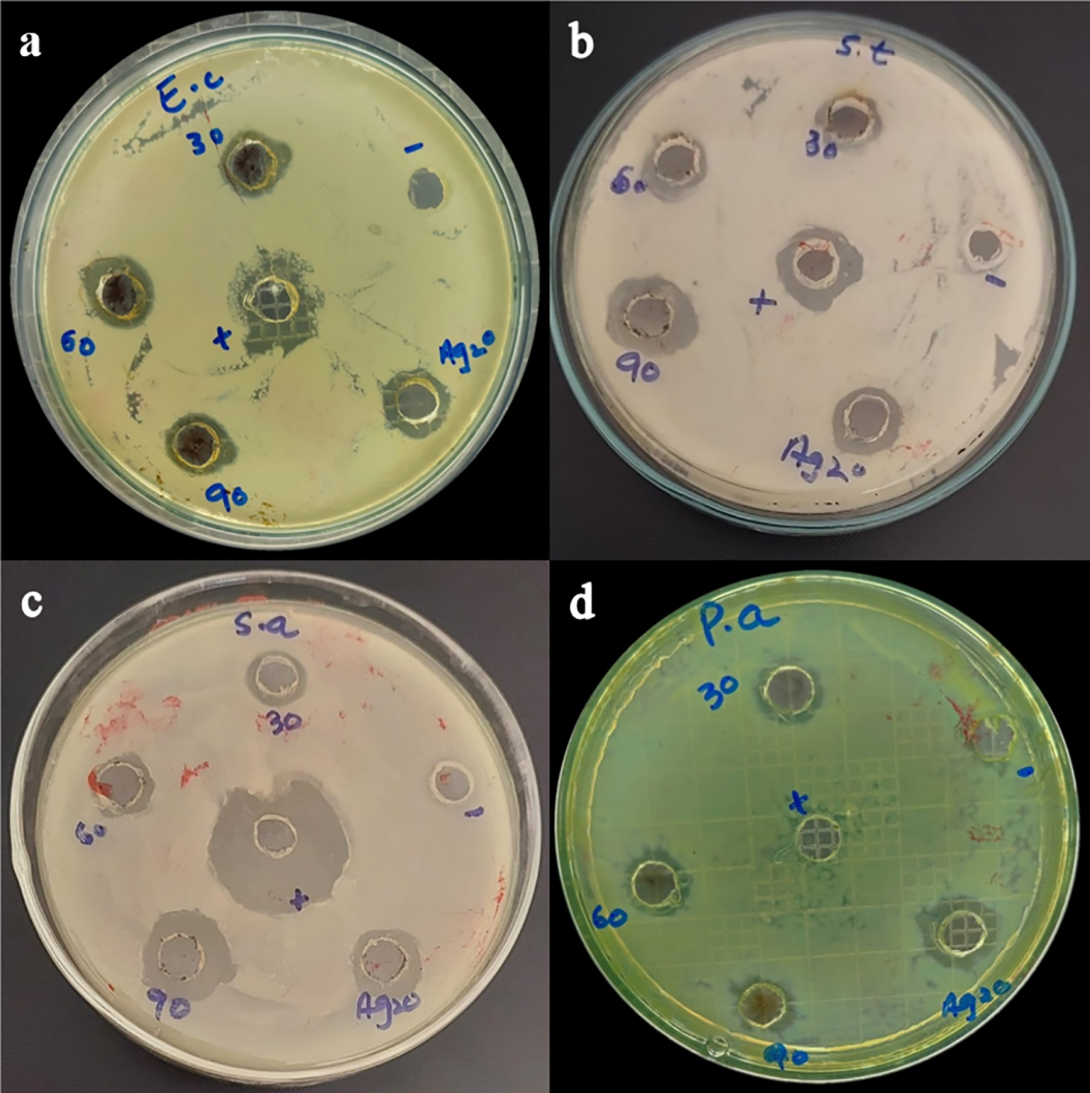
Bacterial zones of inhibition with different concentrations of *Taxus wallichiana* Ag NPs. (A) *Escherichia coli*, (B) *Salmonella typhi*, (C) *Staphylococcus aureus*, (D) *Pseudomonas aeruginosa*.

Based on our results, *Taxus wallichiana* leaf based Ag NPs were found to be significant effective against *E. coli* and *S. typhi* (Fig. 7) where these nanoparticles gave maximum antibacterial activity against *S. typhi* with 19 mm zone of inhibition with 90 ug/well concertation compared to 30ug/well Ampicillin based on statistical analysis (R Core Team, 2022). It was also observed that *S. aureus* gave a maximum zone of inhibition of 16 mm with 90 ug/well concentration which indicated that *Taxus wallichiana* leaf extract Ag NPs works equally well for both gram negative and gram-positive bacteria. A different study based on *Taxus yunnanensis* Ag NPs gave significant antibacterial activity against clinical bacteria strains of *S. aureus*, *B. subtilis*, *E. coli* and *S. paratyphi* B that was attributed to combined effect of silver and plant-based callus extract (*Xia, Ma & Wang, 2016)*. The cell wall of gram-negative bacteria is composed of lipopolysaccharide in addition to peptidoglycan whereas gram positive bacteria cell wall mainly consists of peptidoglycan. Similar observations for both types of bacteria may be due to the adsorbed bioactive compounds on Ag NPs surfaces and Ag NPs large surface to small size ratio to cause cell wall damage. The key underlying mechanism to induce toxic effects as reported by other researchers is the production of reactive oxygen species (ROS) such as superoxide radical (O_2_^−•^), hydroxyl radical (OH^−•^) and hydrogen peroxide (H_2_O_2_) once these Ag NPs are inside the cell. Excessive ROS disrupt a number of biologically important life process, such as fatty acids radical generation in lipid bilayer resulting in lipid peroxidation, protein-enzymes inactivation and their interference with DNA molecule that ultimately leads to bacterial cell death (Fig. 8; Park et al., 2009).

### Effect of silver nanoparticles on cell viability and morphology

Taxol is a major therapeutic compound extracted from *Taxus wallichiana* that belongs to family of diterpenes and is used to treat various sort of cancers. *Taxus wallichiana* is regarded as an endangered tree species by IUCN due to increased demands for Taxol, therefore alternate effective approaches specially based on silver nanoparticles for effective drug delivery can be utilized to compensate for it’s over exploitation (Sharma & Garg, 2015).

Anticancer activity of silver nanoparticles extracted from leaves of *Taxus wallichiana* was performed against U251 cells, derived from a human malignant GBM. It is one of the most aggressive forms of brain cancers and the current therapeutic effects of the approved drugs are not satisfactory especially in terms of resistance development. The major reason for developing resistance and not having the desired therapeutic effects is the presence of blood brain barrier (BBB). There is growing evidence that nano carrier-based therapeutics is a promising approach for the treatment of brain cancer. Thus, based on this, our study is the first novel therapeutic strategy based on Ag NPs adsorbed anticancer compounds (leaf extract of *Taxus wallichiana*) against brain cancer was tested in current study using brain cancer cell line U251.

**Figure 9:**
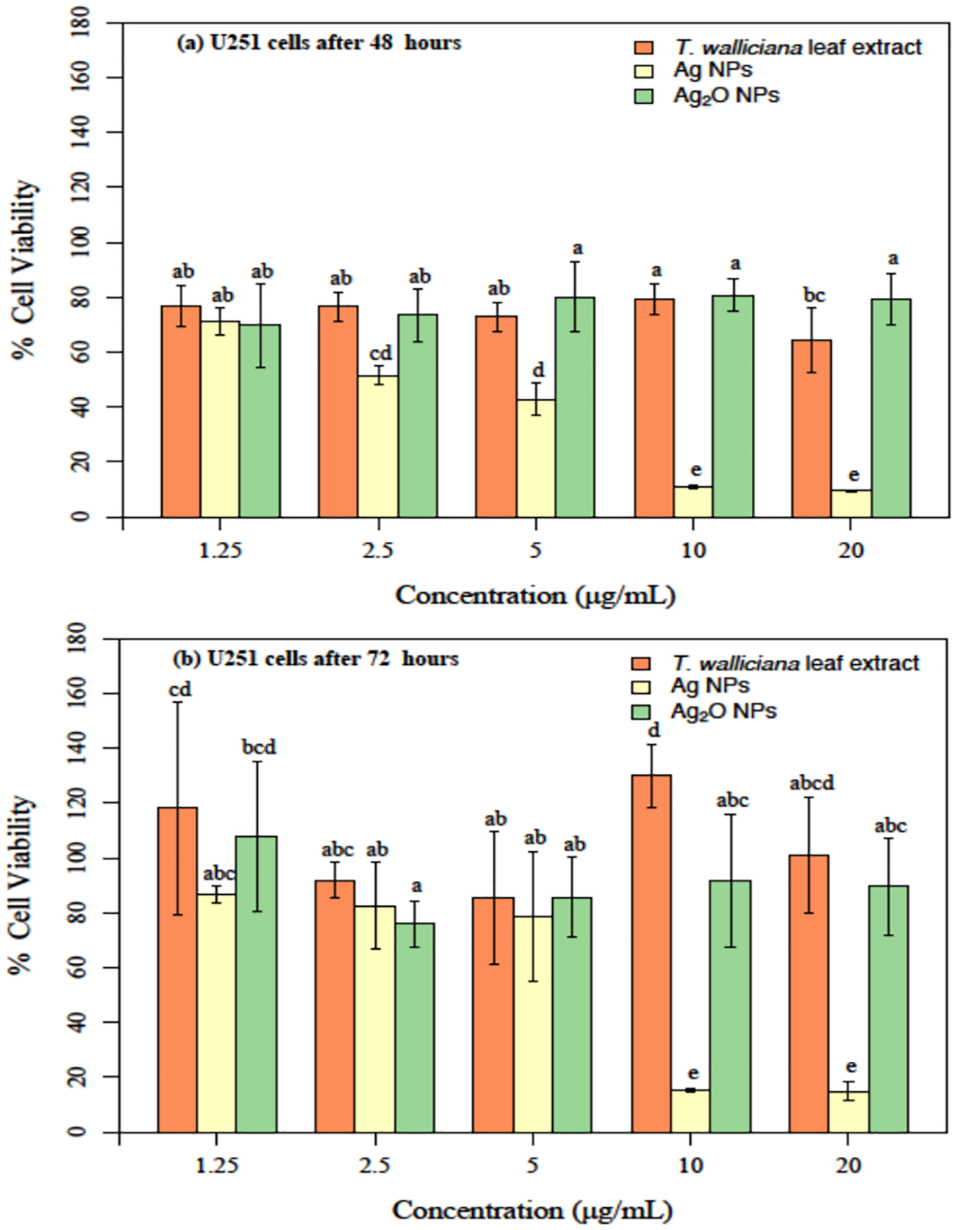
U251 cells after incubation with different concentrations. (A) *Taxus wallichiana*, (B) Ag NPs, (C) Ag_2_O NPs after 48- and 72-h incubation, each with three replicates. Statistical analysis was performed based on ANOVA and the mean values was compared by performing Fisher LSD test using R Program. Mean values are shown for each treatment along with + SE followed by different letters that are significantly different at *p* ≤ 0.05.

The cytotoxicity or anticancer activity indicate that after 48 h of treatment with different formulations, Ag NPs showed a significantly better cytotoxicity as compared to leaf extract and Ag_2_O NPs (Fig. 9A). Significant results were obtained with 2.5 and 5 ug/mL of Ag NPs with 42% and 50% cell viability followed by 10% at 10 and 20 ug/mL Ag NPs concentrations based on two-way ANOVA followed by LSD at 0.05 significance level (R Core Team, 2022). This indicated that cytotoxicity or anticancer activity against U251 cells increased in a dose dependent manner and increasing concentration of Ag NPs resulted in decreased cell viability. The cytotoxic potential were also tested post 72 h of treatment with Ag NPs. Our results showed non-significant difference between treatments at 1.25, 2.5 and 5 ug/mL concentrations of Ag NPs however the cytotoxic potential of the formulation decreased significantly at 10 and 20 ug/mL of Ag NPs as compared to Ag_2_O NPs and *Taxus wallichiana* leaf extract based on statistical analysis (Fig. 9B; R Core Team, 2022).

**Figure 10:**
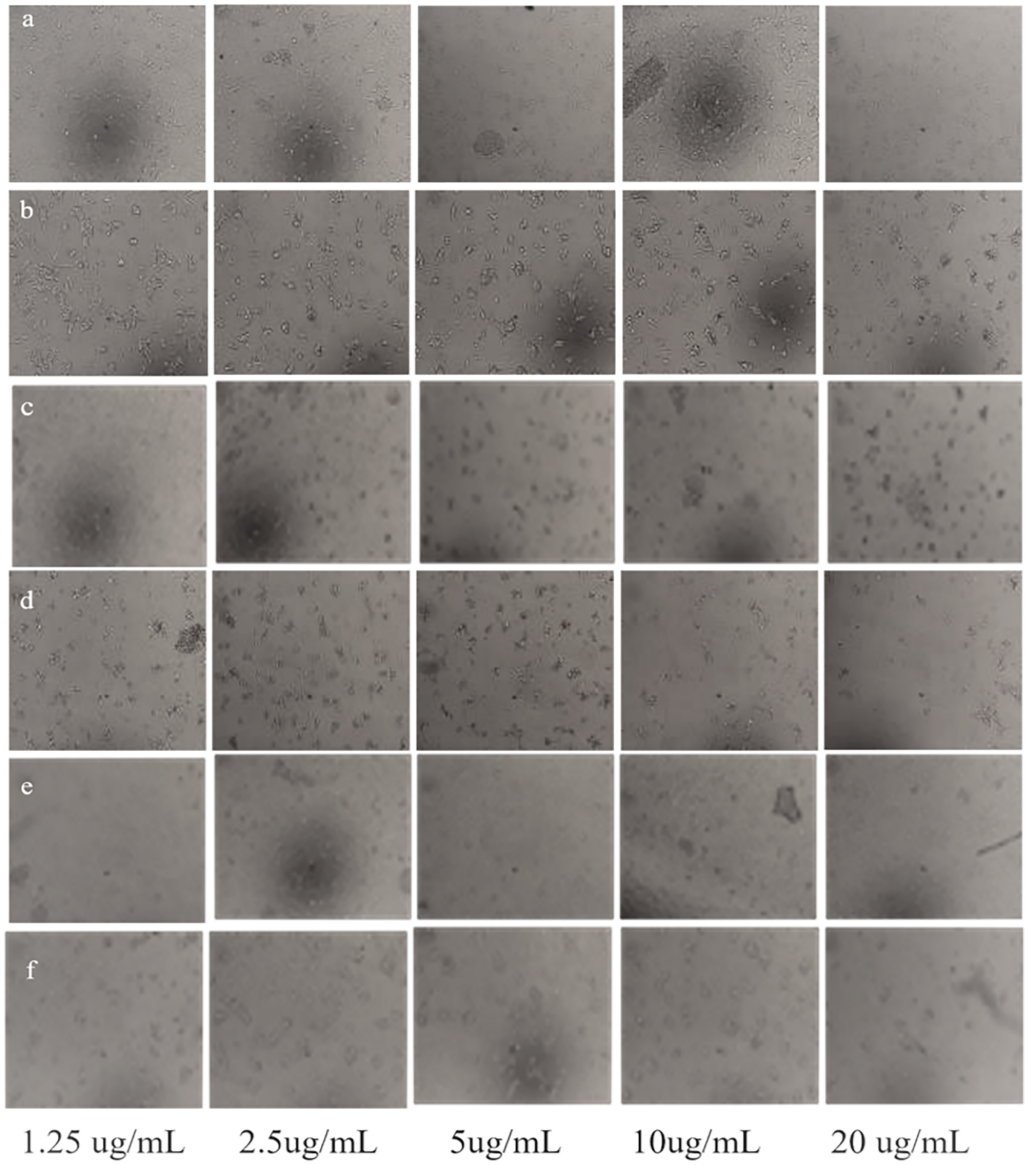
Morphological changes of U251 cancer cells after incubation with *Taxus wallichiana* leaf extract. (A) 48 and (B) 72 hours treated cells; U251 cells after incubation with Ag NPs (C) 48 and (D) 72 h treated cell; U251 cells after incubation with Ag_2_O NPs (E) 48 and (F) 72 h treated cells.

Overall, our results are indicative of the fact that *Taxus wallichiana* Ag NPs can be a potential anticancer therapeutic targeted against brain cancers. Similarly, cell morphologies after the treatment of cells with Ag NPs showed clumping together and cells death as a sign of cytotoxicity with increasing Ag NPs concentrations in a time dependent manner at 48 and 72 h of incubation (Fig. 10).

Ag NPs from *Taxus brevifolia* extract showed cytotoxic effect on human breast cancer cell line MCF-7 with 25 mM that showed 75% mortality rate (Sarli, Kalani & Moradi, 2020). Silver nanoparticles synthesized with *Taxus baccata* extract showed Caov-4 cells mortality up to 50% after 48 h with 2.5 ug/mL concentration and complete mortality with 10 μg/mL concentrations Our results also indicate 50% mortality with 2.5 μg/mL and maximum mortality with 10 μg/mL after 48 h incubation. Although complete cell mortality was observed after 72 h similar to *Taxus baccata* but with different concentrations (Kajani et al., 2016). These somewhat similar findings may be due to intrinsic features such as related metabolites associated with Taxus tree species (Sharma & Garg, 2015). Interestingly, a recent report showed that paclitaxel (brand name Taxol found in *Taxus wallichiana*) loaded Ag_2_O NPs decreased tumor growth of GMB xenografts compared to normal control mice (Chen & Wen, 2022). Similarly, the expression of Beclin1, LC3I, LC3II and glutathione peroxidase 4 (GPX4), marker proteins associated with autophagy induced cell ferroptosis increased significantly in U251 cells treated with paclitaxel Ag_2_O NPs (Chen & Wen, 2022; Wen et al., 2021). Autophagy results in the degradation of iron coated nanoparticles that release iron ions coated with metabolites such as diterpenes and flavonoids causing synergistic effect, that leads to ROS production and eventually causing ferroptosis of GBM cancer cells. This could also be in our case with *Taxus wallichiana* coated Ag NPs treated U251 cancer cells which requires further study.

## Conclusions

In the current study, silver nanoparticles were synthesized using *Taxus wallichiana* leaf extract. Ag NPs showed characteristic absorbance peak at 410 nm. FTIR analysis revealed functional groups of biological origin and XRD and SEM confirmed the average crystallite size of 29 mm with face centered cubic and non-spherical structure. Ag NPs were found effective against both gram-positive and gram-negative *S. typhi* and *S. aureus*. Significant cytotoxic activity was observed with silver nanoparticles against U251 brain cancer cells that resulted in ferroptosis. This report shows for the first time *Taxus wallichiana* leaf-based Ag NPs that could be as a potential drug against glioblastoma. This further requires targeted drug approach and biological pathway elucidation for their successful implementation in cancer therapeutics.

## Supplemental Information

10.7717/peerj.14391/supp-1 Supplemental Information 1 Raw Data for antibacterial activity.

10.7717/peerj.14391/supp-2 Supplemental Information 2 Raw Data U251 anticancer activity with Ag NPs and Ag_2_O NPs.

10.7717/peerj.14391/supp-3 Supplemental Information 3 FTIR data for Ag NPs and Ag_2_O NPs.

10.7717/peerj.14391/supp-4 Supplemental Information 4 Antibacterial activity ANOVA LSD.

10.7717/peerj.14391/supp-5 Supplemental Information 5 One-way and Two-way ANOVA with LSD for antibacterial and anticancer activity.

10.7717/peerj.14391/supp-6 Supplemental Information 6 Supplementary Table 2 Statistical analysis of data recorded after 48 and 72 h after treating U251 cells with *T.wallichiana* leaf extract, AgNPs and Ag_2_O NPs. Statistical analysis was performed based on ANOVA and the mean values was compared by performing Fisher LSD test using R Program. Mean values are shown for each treatment along with + SE followed by different letters that are significantly different at *p* ≤ 0.05

10.7717/peerj.14391/supp-7 Supplemental Information 7 U251 Cell line for anti cancer activity.

## Additional Information and Declarations

### Data Availability

The following information was supplied regarding data availability: The raw data is available in the Supplemental Files.
