# Supplementary material for: Augmented anticancer effect and antibacterial activity of silver nanoparticles synthesized by using Taxus wallichiana leaf extract
Source: PeerJ. 2022 Nov 23;10:e14391. doi: 10.7717/peerj.14391 (PMC9700453; doi:10.7717/peerj.14391)
Supplement: Supplemental Information 4 [file peerj-10-14391-s004.pdf]

E. coli

```
  Df Sum Sq Mean Sq F value   Pr(>F)
df1$Treatment  4  68.83  17.208   36.88 5.86e-06
Residuals     10   4.67   0.467

df1$Treatment ***
Residuals
---
Signif. codes:
0 '***' 0.001 '**' 0.01 '*' 0.05 '.' 0.1 ' ' 1
$statistics
    MSerror Df Mean       CV  t.value      LSD
  0.4666667 10 13.5 5.060223 2.228139 1.242796

$parameters
        test p.ajusted        name.t ntr alpha
  Fisher-LSD      none df1$Treatment   5  0.05

$means
         df1$Ecoli       std r      LCL      UCL
Ag2O      13.33333 0.2886751 3 12.45454 14.21212
AgNP30    11.83333 0.2886751 3 10.95454 12.71212
AgNP60    12.16667 0.2886751 3 11.28788 13.04546
AgNP90    12.50000 1.3228757 3 11.62121 13.37879
Positive  17.66667 0.5773503 3 16.78788 18.54546
          Min  Max   Q25  Q50   Q75
Ag2O     13.0 13.5 13.25 13.5 13.50
AgNP30   11.5 12.0 11.75 12.0 12.00
AgNP60   12.0 12.5 12.00 12.0 12.25
AgNP90   11.5 14.0 11.75 12.0 13.00
Positive 17.0 18.0 17.50 18.0 18.00

$comparison
NULL

$groups
         df1$Ecoli groups
Positive  17.66667      a
Ag2O      13.33333      b
AgNP90    12.50000     bc
AgNP60    12.16667     bc
AgNP30    11.83333      c

attr(,"class")
[1] "group"
```

S. typhi

```
              Df Sum Sq Mean Sq F value Pr(>F)  
df1$Treatment  4 116.77  29.192   5.197 0.0158 *
Residuals     10  56.17   5.617                 
---
Signif. codes:  
0 '***' 0.001 '**' 0.01 '*' 0.05 '.' 0.1 ' ' 1
$statistics
   MSerror Df     Mean       CV  t.value
  5.616667 10 13.93333 17.00922 2.228139
       LSD
  4.311575

$parameters
        test p.ajusted        name.t ntr alpha
  Fisher-LSD      none df1$Treatment   5  0.05

$means
         df1$Styphi       std r       LCL
Ag2O       12.33333 0.2886751 3  9.284589
AgNP30     11.33333 1.1547005 3  8.284589
AgNP60     12.50000 0.5000000 3  9.451256
AgNP90     19.16667 5.1071845 3 16.117923
Positive   14.33333 0.5773503 3 11.284589
              UCL  Min  Max   Q25  Q50   Q75
Ag2O     15.38208 12.0 12.5 12.25 12.5 12.50
AgNP30   14.38208 10.0 12.0 11.00 12.0 12.00
AgNP60   15.54874 12.0 13.0 12.25 12.5 12.75
AgNP90   22.21541 15.5 25.0 16.25 17.0 21.00
Positive 17.38208 14.0 15.0 14.00 14.0 14.50

$comparison
NULL

$groups
         df1$Styphi groups
AgNP90     19.16667      a
Positive   14.33333      b
AgNP60     12.50000      b
Ag2O       12.33333      b
AgNP30     11.33333      b

attr(,"class")
[1] "group"
```

S. aureus

```
              Df Sum Sq Mean Sq F value   Pr(>F)
df1$Treatment  4  197.1   49.28   19.71 9.82e-05
Residuals     10   25.0    2.50

df1$Treatment ***
Residuals
---
Signif. codes:
0 ‘***’ 0.001 ‘**’ 0.01 ‘*’ 0.05 ‘.’ 0.1 ‘ ’ 1
$statistics
  MSerror Df Mean       CV  t.value      LSD
      2.5 10 14.6 10.82972 2.228139 2.876515

$parameters
        test p.ajusted        name.t ntr alpha
  Fisher-LSD      none df1$Treatment   5  0.05

$means
         df1$Saureus       std r       LCL      UCL
Ag2O        12.33333 0.5773503 3 10.299330 14.36734
AgNP30      11.83333 0.7637626 3  9.799330 13.86734
AgNP60      12.00000 0.8660254 3  9.965997 14.03400
AgNP90      15.50000 1.3228757 3 13.465997 17.53400
Positive    21.33333 3.0138569 3 19.299330 23.36734
          Min  Max   Q25  Q50   Q75
Ag2O     12.0 13.0 12.00 12.0 12.50
AgNP30   11.0 12.5 11.50 12.0 12.25
AgNP60   11.0 12.5 11.75 12.5 12.50
AgNP90   14.5 17.0 14.75 15.0 16.00
Positive 18.5 24.5 19.75 21.0 22.75

$comparison
NULL

$groups
         df1$Saureus groups
Positive    21.33333      a
AgNP90      15.50000      b
Ag2O        12.33333      c
AgNP60      12.00000      c
AgNP30      11.83333      c

attr(,"class")
[1] "group"
```

P. aeruginosa

```
              Df Sum Sq Mean Sq F value   Pr(>F)
df1$Treatment  4  378.6   94.64   227.1 9.09e-10
Residuals     10    4.2    0.42

df1$Treatment ***
Residuals
---
Signif. codes:
0 '***' 0.001 '**' 0.01 '*' 0.05 '.' 0.1 ' ' 1
$statistics
    MSerror Df     Mean       CV  t.value
  0.4166667 10 10.03333 6.433527 2.228139
       LSD
  1.174332

$parameters
        test p.ajusted        name.t ntr alpha
  Fisher-LSD      none df1$Treatment   5  0.05

$means
         df1$Paeruginosa       std r        LCL
Ag2O            13.00000 0.0000000 3 12.1696217
AgNP30          12.50000 0.5000000 3 11.6696217
AgNP60          12.16667 0.2886751 3 11.3362883
AgNP90          12.50000 1.3228757 3 11.6696217
Positive         0.00000 0.0000000 3 -0.8303783
                UCL Min  Max   Q25  Q50   Q75
Ag2O     13.8303783  13 13.0 13.00 13.0 13.00
AgNP30   13.3303783  12 13.0 12.25 12.5 12.75
AgNP60   12.9970450  12 12.5 12.00 12.0 12.25
AgNP90   13.3303783  11 13.5 12.00 13.0 13.25
Positive  0.8303783   0  0.0  0.00  0.0  0.00

$comparison
NULL

$groups
         df1$Paeruginosa groups
Ag2O            13.00000      a
AgNP30          12.50000      a
AgNP90          12.50000      a
AgNP60          12.16667      a
Positive         0.00000      b

attr(,"class")
[1] "group"
```
