# Supplementary material for: Augmented anticancer effect and antibacterial activity of silver nanoparticles synthesized by using Taxus wallichiana leaf extract
Source: PeerJ. 2022 Nov 23;10:e14391. doi: 10.7717/peerj.14391 (PMC9700453; doi:10.7717/peerj.14391)
Supplement: Supplemental Information 5 [file peerj-10-14391-s005.pdf]

Fisher-LSD

48 hours Taxus wallichiana Ag NPs and Ag_2O NPs

```
                    Df Sum Sq Mean Sq F value   Pr(>F)    
df1$type             4   2633     658   10.39 2.11e-05 ***
df1$sample           2  14618    7309  115.40 8.17e-15 ***
df1$type:df1$sample  8   6632     829   13.09 6.65e-08 ***
Residuals           30   1900      63                     
---
Signif. codes:  0 '***' 0.001 '**' 0.01 '*' 0.05 '.' 0.1 ' ' 1

  Posthoc multiple comparisons of means : Fisher LSD 
    95% family-wise confidence level

$`df1$type`
          diff     lwr.ci      upr.ci    pval    
2-1  -5.325811 -12.987633   2.3360102 0.16603    
3-1  -7.261341 -14.923162   0.4004805 0.06240 .  
4-1 -15.642131 -23.303952  -7.9803097 0.00024 ***
5-1 -21.461139 -29.122960 -13.7993172 3.1e-06 ***
3-2  -1.935530  -9.597351   5.7262917 0.60969    
4-2 -10.316320 -17.978141  -2.6544985 0.01000 *  
5-2 -16.135327 -23.797149  -8.4735060 0.00017 ***
4-3  -8.380790 -16.042612  -0.7189688 0.03309 *  
5-3 -14.199798 -21.861619  -6.5379764 0.00069 ***
5-4  -5.819008 -13.480829   1.8428138 0.13137    

$`df1$sample`
          diff     lwr.ci     upr.ci    pval    
B-A -36.770451 -42.705272 -30.835629 1.5e-13 ***
C-A   2.773963  -3.160858   8.708785  0.3474    
C-B  39.544414  33.609593  45.479236 2.3e-14 ***

$`df1$type:df1$sample`
               diff     lwr.ci      upr.ci    pval    
2:A-1:A  -0.1862792 -13.456943  13.0843847 0.97732    
3:A-1:A  -3.8533184 -17.123982   9.4173455 0.55762    
4:A-1:A   2.2992176 -10.971446  15.5698815 0.72594    
5:A-1:A -12.4540955 -25.724759   0.8165684 0.06486 .  
1:B-1:A  -5.6203098 -18.890974   7.6503542 0.39394    
2:B-1:A -25.2275267 -38.498191 -11.9568628 0.00053 ***
3:B-1:A -33.8974932 -47.168157 -20.6268293 1.3e-05 ***
4:B-1:A -65.9800947 -79.250759 -52.7094308 3.2e-11 ***
5:B-1:A -67.3213050 -80.591969 -54.0506411 2.0e-11 ***
1:C-1:A  -7.0786098 -20.349274   6.1920541 0.28467    
2:C-1:A  -3.2625472 -16.533211  10.0081167 0.61927    
3:C-1:A   3.2678695 -10.002794  16.5385334 0.61870    
4:C-1:A   4.0555644  -9.215099  17.3262283 0.53726    
5:C-1:A   2.6930651 -10.577599  15.9637290 0.68150    
3:A-2:A  -3.6670392 -16.937703   9.6036247 0.57672    
4:A-2:A   2.4854968 -10.785167  15.7561607 0.70479    
5:A-2:A -12.2678163 -25.538480   1.0028476 0.06874 .  
1:B-2:A  -5.4340305 -18.704694   7.8366334 0.40962    
2:B-2:A -25.0412475 -38.311911 -11.7705836 0.00057 ***
```

3:B-2:A	-33.7112140	-46.981878	-20.4405501	1.4e-05	***
4:B-2:A	-65.7938155	-79.064479	-52.5231516	3.4e-11	***
5:B-2:A	-67.1350258	-80.405690	-53.8643619	2.1e-11	***
1:C-2:A	-6.8923306	-20.162995	6.3783333	0.29730	
2:C-2:A	-3.0762680	-16.346932	10.1943959	0.63934	
3:C-2:A	3.4541487	-9.816515	16.7248126	0.59894	
4:C-2:A	4.2418436	-9.028820	17.5125075	0.51886	
5:C-2:A	2.8793443	-10.391320	16.1500082	0.66086	
4:A-3:A	6.1525361	-7.118128	19.4232000	0.35129	
5:A-3:A	-8.6007771	-21.871441	4.6698869	0.19563	
1:B-3:A	-1.7669913	-15.037655	11.5036726	0.78754	
2:B-3:A	-21.3742083	-34.644872	-8.1035444	0.00257	**
3:B-3:A	-30.0441748	-43.314839	-16.7735109	6.7e-05	***
4:B-3:A	-62.1267763	-75.397440	-48.8561124	1.3e-10	***
5:B-3:A	-63.4679866	-76.738651	-50.1973227	7.9e-11	***
1:C-3:A	-3.2252914	-16.495955	10.0453725	0.62326	
2:C-3:A	0.5907712	-12.679893	13.8614351	0.92816	
3:C-3:A	7.1211879	-6.149476	20.3918518	0.28184	
4:C-3:A	7.9088829	-5.361781	21.1795468	0.23304	
5:C-3:A	6.5463835	-6.724280	19.8170474	0.32178	
5:A-4:A	-14.7533131	-28.023977	-1.4826492	0.03053	*
1:B-4:A	-7.9195274	-21.190191	5.3511365	0.23243	
2:B-4:A	-27.5267444	-40.797408	-14.2560805	0.00020	***
3:B-4:A	-36.1967108	-49.467375	-22.9260469	4.7e-06	***
4:B-4:A	-68.2793124	-81.549976	-55.0086485	1.4e-11	***
5:B-4:A	-69.6205226	-82.891187	-56.3498587	9.0e-12	***
1:C-4:A	-9.3778275	-22.648491	3.8928365	0.15933	
2:C-4:A	-5.5617649	-18.832429	7.7088991	0.39883	
3:C-4:A	0.9686519	-12.302012	14.2393158	0.88250	
4:C-4:A	1.7563468	-11.514317	15.0270107	0.78879	
5:C-4:A	0.3938475	-12.876816	13.6645114	0.95207	
1:B-5:A	6.8337857	-6.436878	20.1044496	0.30135	
2:B-5:A	-12.7734313	-26.044095	0.4972326	0.05864	.
3:B-5:A	-21.4433977	-34.714062	-8.1727338	0.00250	**
4:B-5:A	-53.5259993	-66.796663	-40.2553353	3.4e-09	***
5:B-5:A	-54.8672095	-68.137873	-41.5965456	2.0e-09	***
1:C-5:A	5.3754857	-7.895178	18.6461496	0.41463	
2:C-5:A	9.1915483	-4.079116	22.4622122	0.16750	
3:C-5:A	15.7219650	2.451301	28.9926289	0.02181	*
4:C-5:A	16.5096599	3.238996	29.7803238	0.01647	*
5:C-5:A	15.1471606	1.876497	28.4178245	0.02666	*
2:B-1:B	-19.6072170	-32.877881	-6.3365531	0.00516	**
3:B-1:B	-28.2771835	-41.547847	-15.0065195	0.00014	***
4:B-1:B	-60.3597850	-73.630449	-47.0891211	2.5e-10	***
5:B-1:B	-61.7009953	-74.971659	-48.4303314	1.5e-10	***
1:C-1:B	-1.4583001	-14.728964	11.8123638	0.82395	
2:C-1:B	2.3577625	-10.912901	15.6284264	0.71926	
3:C-1:B	8.8881793	-4.382485	22.1588432	0.18152	
4:C-1:B	9.6758742	-3.594790	22.9465381	0.14691	
5:C-1:B	8.3133749	-4.957289	21.5840388	0.21057	
3:B-2:B	-8.6699665	-21.940630	4.6006974	0.19216	
4:B-2:B	-40.7525680	-54.023232	-27.4819041	6.5e-07	***
5:B-2:B	-42.0937783	-55.364442	-28.8231144	3.7e-07	***
1:C-2:B	18.1489169	4.878253	31.4195808	0.00901	**

2:C-2:B	21.9649795	8.694316	35.2356434	0.00203	**
3:C-2:B	28.4953962	15.224732	41.7660602	0.00013	***
4:C-2:B	29.2830912	16.012427	42.5537551	9.3e-05	***
5:C-2:B	27.9205918	14.649928	41.1912558	0.00017	***
4:B-3:B	-32.0826015	-45.353265	-18.8119376	2.8e-05	***
5:B-3:B	-33.4238118	-46.694476	-20.1531479	1.6e-05	***
1:C-3:B	26.8188834	13.548219	40.0895473	0.00027	***
2:C-3:B	30.6349460	17.364282	43.9056099	5.2e-05	***
3:C-3:B	37.1653627	23.894699	50.4360266	3.1e-06	***
4:C-3:B	37.9530576	24.682394	51.2237215	2.2e-06	***
5:C-3:B	36.5905583	23.319894	49.8612222	3.9e-06	***
5:B-4:B	-1.3412103	-14.611874	11.9294536	0.83787	
1:C-4:B	58.9014849	45.630821	72.1721488	4.3e-10	***
2:C-4:B	62.7175475	49.446884	75.9882114	1.0e-10	***
3:C-4:B	69.2479642	55.977300	82.5186281	1.0e-11	***
4:C-4:B	70.0356592	56.764995	83.3063231	7.8e-12	***
5:C-4:B	68.6731598	55.402496	81.9438237	1.2e-11	***
1:C-5:B	60.2426952	46.972031	73.5133591	2.6e-10	***
2:C-5:B	64.0587578	50.788094	77.3294217	6.4e-11	***
3:C-5:B	70.5891745	57.318511	83.8598384	6.4e-12	***
4:C-5:B	71.3768694	58.106206	84.6475334	4.9e-12	***
5:C-5:B	70.0143701	56.743706	83.2850340	7.8e-12	***
2:C-1:C	3.8160626	-9.454601	17.0867265	0.56142	
3:C-1:C	10.3464793	-2.924185	23.6171432	0.12181	
4:C-1:C	11.1341742	-2.136490	24.4048382	0.09694	.
5:C-1:C	9.7716749	-3.498989	23.0423388	0.14309	
3:C-2:C	6.5304167	-6.740247	19.8010806	0.32294	
4:C-2:C	7.3181117	-5.952552	20.5887756	0.26900	
5:C-2:C	5.9556123	-7.315052	19.2262762	0.36670	
4:C-3:C	0.7876949	-12.482969	14.0583588	0.90432	
5:C-3:C	-0.5748044	-13.845468	12.6958595	0.93010	
5:C-4:C	-1.3624993	-14.633163	11.9081646	0.83533	

Signif. codes: 0 '***' 0.001 '**' 0.01 '*' 0.05 '.' 0.1 ' ' 1

2:A	3:A	4:A	5:A	1:B	2:B	3:B	4:B	5:B	1:C	2:C	3:C	4:C	5:C	1:A
"ab"	"ab"	"a"	"bc"	"ab"	"cd"	"d"	"e"	"e"	"ab"	"ab"	"a"	"a"	"a"	"ab"

72 hours Taxus wallichiana Ag NPs and Ag_2O NPs

	Df	Sum Sq	Mean Sq	F value	Pr(>F)	
df1$type	4	6097	1524	4.158	0.008493	**
df1$sample	2	19523	9762	26.632	2.24e-07	***
df1$type:df1$sample	8	16130	2016	5.501	0.000256	***
Residuals	30	10996	367			

Signif. codes: 0 '***' 0.001 '**' 0.01 '*' 0.05 '.' 0.1 ' ' 1

Posthoc multiple comparisons of means : Fisher LSD

```
    95% family-wise confidence level

$`df1$type`
           diff    lwr.ci     upr.ci    pval    
2-1 -20.8608785 -39.29262  -2.429139 0.02786 *  
3-1 -21.0456259 -39.47737  -2.613886 0.02661 *  
4-1 -25.1902008 -43.62194  -6.758461 0.00905 ** 
5-1 -35.8231945 -54.25493 -17.391455 0.00042 ***
3-2  -0.1847474 -18.61649  18.246992 0.98380    
4-2  -4.3293223 -22.76106  14.102418 0.63492    
5-2 -14.9623160 -33.39406   3.469424 0.10777    
4-3  -4.1445749 -22.57631  14.287165 0.64938    
5-3 -14.7775685 -33.20931   3.654171 0.11200    
5-4 -10.6329937 -29.06473   7.798746 0.24800    

$`df1$sample`
         diff    lwr.ci      upr.ci    pval    
B-A -49.75760 -64.03477 -35.4804382 6.5e-08 ***
C-A -15.10878 -29.38594  -0.8316141  0.0388 *  
C-B  34.64882  20.37166  48.9259884 2.6e-05 ***

$`df1$type:df1$sample`
                 diff      lwr.ci      upr.ci    pval    
2:A-1:A  -26.2430386  -58.167748   5.6816713 0.10358    
3:A-1:A  -32.7136504  -64.638360  -0.7889405 0.04493 *  
4:A-1:A   12.0397452  -19.884965  43.9644551 0.44720    
5:A-1:A  -17.0613139  -48.986024  14.8633960 0.28377    
1:B-1:A  -31.4916463  -63.416356   0.4330636 0.05299 .  
2:B-1:A  -35.7052232  -67.629933  -3.7805133 0.02961 *  
3:B-1:A  -39.5582088  -71.482919  -7.6334989 0.01687 *  
4:B-1:A -102.6884090 -134.613119 -70.7636991 2.9e-07 ***
5:B-1:A -103.3227827 -135.247493 -71.3980728 2.6e-07 ***
1:C-1:A  -10.0097493  -41.934459  21.9149606 0.52681    
2:C-1:A  -42.1357693  -74.060479 -10.2110594 0.01141 *  
3:C-1:A  -32.3664143  -64.291124  -0.4417043 0.04710 *  
4:C-1:A  -26.4233343  -58.348044   5.5013756 0.10133    
5:C-1:A  -28.5868825  -60.511592   3.3378274 0.07740 .  
3:A-2:A   -6.4706118  -38.395322  25.4540981 0.68187    
4:A-2:A   38.2827838    6.358074  70.2074937 0.02038 *  
5:A-2:A    9.1817247  -22.742985  41.1064346 0.56135    
1:B-2:A   -5.2486077  -37.173318  26.6761022 0.73939    
2:B-2:A   -9.4621846  -41.386895  22.4625253 0.54952    
3:B-2:A  -13.3151702  -45.239880  18.6095397 0.40108    
4:B-2:A  -76.4453704 -108.370080 -44.5206605 3.2e-05 ***
5:B-2:A  -77.0797441 -109.004454 -45.1550342 2.8e-05 ***
1:C-2:A   16.2332893  -15.691421  48.1579992 0.30735    
2:C-2:A  -15.8927307  -47.817441  16.0319792 0.31743    
3:C-2:A   -6.1233757  -38.048086  25.8013342 0.69803    
4:C-2:A   -0.1802957  -32.105006  31.7444142 0.99087    
5:C-2:A   -2.3438439  -34.268554  29.5808660 0.88182    
4:A-3:A   44.7533956   12.828686  76.6781055 0.00758 ** 
5:A-3:A   15.6523365  -16.272373  47.5770464 0.32469    
1:B-3:A    1.2220041  -30.702706  33.1467140 0.93821    
2:B-3:A   -2.9915728  -34.916283  28.9331371 0.84952    
```

3:B-3:A	-6.8445584	-38.769268	25.0801515	0.66463	
4:B-3:A	-69.9747586	-101.899468	-38.0500487	0.00010	***
5:B-3:A	-70.6091323	-102.533842	-38.6844224	9.1e-05	***
1:C-3:A	22.7039011	-9.220809	54.6286110	0.15677	
2:C-3:A	-9.4221189	-41.346829	22.5025910	0.55121	
3:C-3:A	0.3472361	-31.577474	32.2719461	0.98242	
4:C-3:A	6.2903161	-25.634394	38.2150260	0.69024	
5:C-3:A	4.1267679	-27.797942	36.0514778	0.79359	
5:A-4:A	-29.1010591	-61.025769	2.8236508	0.07248	.
1:B-4:A	-43.5313915	-75.456101	-11.6066816	0.00919	**
2:B-4:A	-47.7449684	-79.669678	-15.8202585	0.00470	**
3:B-4:A	-51.5979540	-83.522664	-19.6732441	0.00249	**
4:B-4:A	-114.7281542	-146.652864	-82.8034443	3.6e-08	***
5:B-4:A	-115.3625279	-147.287238	-83.4378180	3.2e-08	***
1:C-4:A	-22.0494945	-53.974204	9.8752154	0.16867	
2:C-4:A	-54.1755145	-86.100224	-22.2508046	0.00162	**
3:C-4:A	-44.4061594	-76.330869	-12.4814495	0.00801	**
4:C-4:A	-38.4630795	-70.387789	-6.5383695	0.01985	*
5:C-4:A	-40.6266277	-72.551338	-8.7019178	0.01436	*
1:B-5:A	-14.4303324	-46.355042	17.4943775	0.36330	
2:B-5:A	-18.6439093	-50.568619	13.2808006	0.24234	
3:B-5:A	-22.4968949	-54.421605	9.4278150	0.16046	
4:B-5:A	-85.6270951	-117.551805	-53.7023852	6.0e-06	***
5:B-5:A	-86.2614688	-118.186179	-54.3367589	5.4e-06	***
1:C-5:A	7.0515646	-24.873145	38.9762745	0.65516	
2:C-5:A	-25.0744554	-56.999165	6.8502545	0.11918	
3:C-5:A	-15.3051003	-47.229810	16.6196096	0.33537	
4:C-5:A	-9.3620204	-41.286730	22.5626895	0.55373	
5:C-5:A	-11.5255686	-43.450278	20.3991413	0.46666	
2:B-1:B	-4.2135769	-36.138287	27.7111330	0.78935	
3:B-1:B	-8.0665625	-39.991272	23.8581474	0.60962	
4:B-1:B	-71.1967627	-103.121473	-39.2720528	8.2e-05	***
5:B-1:B	-71.8311364	-103.755846	-39.9064265	7.3e-05	***
1:C-1:B	21.4818970	-10.442813	53.4066069	0.17955	
2:C-1:B	-10.6441230	-42.568833	21.2805869	0.50114	
3:C-1:B	-0.8747680	-32.799478	31.0499420	0.95574	
4:C-1:B	5.0683120	-26.856398	36.9930219	0.74802	
5:C-1:B	2.9047638	-29.019946	34.8294737	0.85383	
3:B-2:B	-3.8529856	-35.777695	28.0717243	0.80699	
4:B-2:B	-66.9831858	-98.907896	-35.0584759	0.00017	***
5:B-2:B	-67.6175595	-99.542269	-35.6928496	0.00015	***
1:C-2:B	25.6954739	-6.229236	57.6201838	0.11066	
2:C-2:B	-6.4305461	-38.355256	25.4941638	0.68372	
3:C-2:B	3.3388090	-28.585901	35.2635189	0.83231	
4:C-2:B	9.2818889	-22.642821	41.2065989	0.55711	
5:C-2:B	7.1183407	-24.806369	39.0430506	0.65212	
4:B-3:B	-63.1302002	-95.054910	-31.2054903	0.00034	***
5:B-3:B	-63.7645739	-95.689284	-31.8398640	0.00031	***
1:C-3:B	29.5484595	-2.376250	61.4731694	0.06842	.
2:C-3:B	-2.5775605	-34.502270	29.3471494	0.87014	
3:C-3:B	7.1917945	-24.732915	39.1165045	0.64878	
4:C-3:B	13.1348745	-18.789835	45.0595844	0.40741	
5:C-3:B	10.9713263	-20.953384	42.8960362	0.48818	
5:B-4:B	-0.6343737	-32.559084	31.2903362	0.96790	

```
1:C-4:B   92.6786597   60.753950 124.6033696 1.7e-06 ***
2:C-4:B   60.5526397   28.627930  92.4773496 0.00054 ***
3:C-4:B   70.3219947   38.397285 102.2467046 9.6e-05 ***
4:C-4:B   76.2650747   44.340365 108.1897846 3.3e-05 ***
5:C-4:B   74.1015265   42.176817 106.0262364 4.8e-05 ***
1:C-5:B   93.3130334   61.388323 125.2377433 1.5e-06 ***
2:C-5:B   61.1870134   29.262303  93.1117233 0.00048 ***
3:C-5:B   70.9563684   39.031659 102.8810783 8.5e-05 ***
4:C-5:B   76.8994484   44.974739 108.8241583 2.9e-05 ***
5:C-5:B   74.7359002   42.811190 106.6606101 4.3e-05 ***
2:C-1:C  -32.1260200  -64.050730  -0.2013101 0.04866 *
3:C-1:C  -22.3566649  -54.281375   9.5680450 0.16300
4:C-1:C  -16.4135850  -48.338295  15.5111249 0.30210
5:C-1:C  -18.5771332  -50.501843  13.3475767 0.24400
3:C-2:C    9.7693551  -22.155355  41.6940650 0.53672
4:C-2:C   15.7124350  -16.212275  47.6371450 0.32286
5:C-2:C   13.5488868  -18.375823  45.4735967 0.39296
4:C-3:C    5.9430800  -25.981630  37.8677899 0.70648
5:C-3:C    3.7795318  -28.145178  35.7042417 0.81059
5:C-4:C   -2.1635482  -34.088258  29.7611617 0.89084

---
Signif. codes:  0 '***' 0.001 '**' 0.01 '*' 0.05 '.' 0.1 ' ' 1

   2:A    3:A    4:A    5:A    1:B    2:B    3:B    4:B    5:B    1:C 
2:C 
 "abc"   "ab"    "d" "abcd"  "abc"   "ab"   "ab"    "e"    "e"  "bcd" 
"a" 
   3:C    4:C    5:C    1:A 
  "ab"  "abc"  "abc"   "cd" 
```
