# Supplementary material for: Augmented anticancer effect and antibacterial activity of silver nanoparticles synthesized by using Taxus wallichiana leaf extract
Source: PeerJ. 2022 Nov 23;10:e14391. doi: 10.7717/peerj.14391 (PMC9700453; doi:10.7717/peerj.14391)
Supplement: Supplemental Information 6 — Statistical analysis was performed based on ANOVA and the mean values was compared by performing Fisher LSD test using R Program. Mean values are shown for each treatment along with + SE followed by different letters that are significantly different at p ≤ 0.05 [file peerj-10-14391-s006.docx]

| S. No. | Concentrations of T. *wallichiana* Leaf extract AgNPs and Ag_2_O | U251 % cells viability with *T. wallichiana* extract | | % cells viability with *T. wallichiana* AgNPs | | % cells viability with *T. wallichiana* Ag_2_ONPs | |
| --- | --- | --- | --- | --- | --- | --- | --- |
|  |  | **48hrs** | **72hrs** | **48hrs** | **72hrs** | **48hrs** | **72hrs** |
| 1. | 1.25 ug/ml | 79.0+ 7.74a | 130+  38.7a | 71.1+ 4.87 a | 86.6+ 3.21a | 80.7+ 15.1a | 108+ 27.3a |
| 2. | 2.5 ug/ml | 76.7+ 5.40ab | 118+  6.3a | 51.5+  3.2 6b | 82.4+ 15.8a | 80.0+ 9.49a | 91.6+ 8.37a |
| 3. | 5.0 ug/ml | 76.5+ 5.33ab | 101+  24.3a | 42.8+ 5.74b | 78.5+ 23.8a | 79.4+ 12.6a | 89.5+ 14.3a |
| 4. | 10 ug/ml | 72.8+ 5.57ab | 91+  11.5a | 10.7+ 0.75c | 15.4+  0.6b | 73.4+ 5.92a | 85.7+ 24.4a |
| 5. | 20 ug/ml | 64.2+ 11.5b | 85+  21.3a | 9.4+ 0.40c | 14.7+  3.4b | 69.6+  9.26a | 75.9+ 17.7a |
| 7. | F-Value | 2.39 | 0.007 | 167.7 | 35.8 | 2.21 | 0.33 |

**Supplementary Table 2 Statistical analysis of data recorded after 48 and 72 hours after treating U251 cells with *T.wallichiana* leaf extract, AgNPs and Ag_2_O NPs**. Statistical analysis was performed based on ANOVA and the mean values was compared by performing Fisher LSD test using R Program. Mean values are shown for each treatment along with + SE followed by different letters that are significantly different at P ≤ 0.05.
