# Supplementary material for: Augmented anticancer effect and antibacterial activity of silver nanoparticles synthesized by using Taxus wallichiana leaf extract
Source: PeerJ. 2022 Nov 23;10:e14391. doi: 10.7717/peerj.14391 (PMC9700453; doi:10.7717/peerj.14391)
Supplement: Supplemental Information 7 [file peerj-10-14391-s007.docx]

Glioblastoma multiform cell line was a kind gift from Prof. Norah Defamie. Pôle Biologie Sante Université de Poitiers, France.
